# Introduction of a near peer surgical tutor program for final year medical students

**DOI:** 10.15694/mep.2017.000162

**Published:** 2017-09-18

**Authors:** Justin C. Tse, Corinne C. Tey, Lauren M. Sanders

**Affiliations:** 1 University of Melbourne; 2 University of Melbourne & St Vincent's Hospital

**Keywords:** Near Peer Teaching

## Abstract

This article was migrated. The article was marked as recommended.

## Letter

Dear Ms Browne & Dr Read,

Thank you for the opportunity to contribute to the Themed Issue: Medical students and postgraduate trainees as medical educators. We recently introduced a near peer surgical tutor program for final year medical students in the MD program at the Melbourne Medical School, The University of Melbourne. The aim of the program was to address the reduced clinical exposure during the students’ dedicated research semester whilst enhancing the teaching skills of junior surgical trainees. To follow, we provide an overview of our initial experience with developing and evaluating this program.

Near peer teaching has gained traction in recent times as a formal teaching tool to enhance learning and skills acquisition ( Liew, 2015). As noted in the literature, there is value not only for students but also the tutors that deliver the teaching ( Afghani, 2013; Ramani, 2016). Although under-utilised ( van de Mortel, 2016), near peer teaching is able to provide additional, valuable experience for students and tutors alike, and is worthy of further consideration.

At the Melbourne Medical School, medical students undertake a year-long research subject as part of a four-year program. In the second semester of third year, research tasks are planned alongside clinical commitments. Students are required to devote the entire first semester of final year to their research project, with a completed literature review and journal monograph as assessed outcomes of the semester. Students are encouraged to spend clinical time with research supervisors, but this is not compulsory and not all projects have a clinical component. This leaves students with 16 weeks in their final semester to reacquaint themselves with the clinical environment and prepare for internship.

At St Vincent’s Clinical School, we have devised a hybrid program to ensure that students in the research semester have continued exposure to clinical learning and ward experiences. Taking resource limitations into account, this includes a simulation program with deteriorating patient scenarios and also a surgical hospital medical officer (HMO) near peer teaching program.

The surgical HMO near peer teaching program, which commenced in 2017, was designed to encourage and develop teaching skills in junior trainees, as well as to assist in their own preparation for surgical examinations. Tutors were selected via an application process, with successful candidates required to attend an information session on program arrangements and a training session on teaching and debriefing skills. Peer tutors were assigned a group of 6 final year students with the bedside tutorial schedule determined following discussion between tutors and group leaders. Tutorial content was based around clinical problems commonly encountered by junior doctors including post-operative fever, common fractures and peri-operative management of patients.

The surgical near peer program evaluation was approved by the hospital Quality and Risk Department (QA033/17). On initial program evaluation, both students and tutors have found the experience rewarding. Themes emerging from the feedback suggest that the program has been able to achieve the following:

Students:

• Ability to maintain clinical exposure during a research-focused semester
• Ability to network with trainees who may become their colleagues in future
• Ability to transition from their penultimate, research-focused semester into the pre-internship semester without significant attrition of clinical skills and knowledge

Tutors:

• Ability to hone teaching skills for those interested in a future career in medical education
• Ability to enhance their own clinical knowledge through the process of imparting information and skills to medical students

School:

• In view of limited resources, the near peer teaching program has provided a partial solution to the problem of insufficient clinical contact faced by some of our students during their research intensive semester
• Ability to identify surgical HMO tutors with an interest in medical education and provide training to promote development of tutor teaching skills

Moving forward, further evaluation of the surgical near peer program will be carried out, together with continued development of the tutor training program to ensure that effective teaching is delivered. This continuous improvement approach has been described as key to an effective near peer teaching program ( Chokshi, 2017).

Finally, ongoing research is required to determine best practice for near peer teaching programs that address the needs of students, tutors and program requirements ( Ramani, 2016). There are positive experiences reported from many institutions around the world, and if we can harness near peer teaching within formal program structures, it becomes another valuable vehicle for the delivery of medical education.

Yours sincerely,

A/Prof Justin Tse

Dr Corinne Tey

Dr Lauren Sanders

## Notes On Contributors

A/Prof Justin Tse (MBBS MMed (Research) FRACGP FACHI) is the Director of Medical Student Education (Clinical Dean) at St Vincent’s Clinical School, University of Melbourne. He is a General Practionar and completed his research thesis in Prostate Cancer screening. His research interests include medical education and the increasing role of technology in medical practice.

Dr Corinne Tey (MBBS FRACP GradDipClinEd) is the Clinical Subdean at St Vincent’s Clinical School, University of Melbourne. She practises as a consultant General Physician at St Vincent’s Hospital and acts as educational supervisor for General Medicine interns.

Dr Lauren Sanders (MBBS GradDipIntlHealth PhD FRACP GradCertClinTeach) is the MD Research Project co-ordinator at St Vincent’s Clinical School, University of Melbourne. She is involved in teaching as a Clinical Skills Coach and lecturer for MD2 and MD4 students. She also practises as a consultant Neurologist with clinical and research expertise in Stroke.
