# Supplementary material for: Introduction of a near peer surgical tutor program for final year medical students
Source: MedEdPublish (2016). 2017 Sep 18;6:162. [Version 1] doi: 10.15694/mep.2017.000162 (PMC10885293; doi:10.15694/mep.2017.000162)
Supplement: Peer review history

# Reviewer report: Reviewer response for version 1

**DOI:** 10.21956/mep.19349.r28283

**Published:** 2017-09-28

**Authors:** James Read

**Affiliations:** 1 Plymouth University Peninsula Schools of Medicine and Dentistry

This review has been migrated. The reviewer awarded 4 stars out of 5 Dear A/Prof Tse, Dr Tey and Dr Sanders.Many thanks for sharing your experiences of near-peer education with us as part of this themed edition of MedEdPublish. It is really encouraging to read how peer education can be used to promote ongoing learning and maintaining clinical skills whilst taking a step back from direct patient contact to undertake some research training.It is very positive to read the feedback that you have received from your students and those who have delivered some of the education. I particularly like your point that such an approach can be a very good use of resources, especially as it requires very little in terms of additional facilities.Thank you again for sharing your experiences with us and I wish you the very best of luck with the continued development of this in the future.Best wishes,Jamie Read

Reviewer Expertise:

NA

No decision status is available

# Reviewer report: Reviewer response for version 1

**DOI:** 10.21956/mep.19349.r28284

**Published:** 2017-09-18

**Authors:** John Dent

**Affiliations:** 1 AMEE

This review has been migrated. The reviewer awarded 4 stars out of 5 It is interesting to read this letter describing experiences with near-peer tutoring. I am pleased that the authors have used this opportunity to share their programme and to comment on the early findings.Others starting on a similar journey should find this account both helpful and encouraging.

Reviewer Expertise:

NA

No decision status is available
